# A comprehensive overview of exosome lncRNAs: emerging biomarkers and potential therapeutics in endometriosis

**DOI:** 10.3389/fendo.2023.1199569

**Published:** 2023-06-26

**Authors:** Min Wang, Lianwen Zheng, Ruixin Lin, Shuai Ma, Jiahui Li, Shuli Yang

**Affiliations:** ^1^ Department of Obstetrics and Gynecology, The Second Hospital of Jilin University, Changchun, China; ^2^ Department of Hepato-Biliary-Pancreatic Surgery, The Second Hospital of Jilin University, Changchun, China

**Keywords:** exosome lncRNA, expression, biomarkers, therapeutics, endometriosis

## Abstract

Endometriosis is a gynecological condition that significantly impacting women’s daily lives. In recent years, the incidence of endometriosis has been rising yearly and is now an essential contributor to female infertility. Exosomes are extracellular vesicles (EVs) that carry long noncoding RNA (lncRNA) and shield lncRNA from the outside environment thanks to their vesicle-like structure. The role of exosome-derived lncRNAs in endometriosis is also receiving more study as high-throughput sequencing technology develops. Several lncRNAs with variable expression may be crucial to the emergence and growth of endometriosis. The early diagnosis of endometriosis will be considerably improved by further high specificity and sensitivity Exosome lncRNA screening. Exosomes assist lncRNAs in carrying out their roles, offering a new target for creating endometriosis-specific medications. In order to serve as a reference for clinical research on the pathogenesis, diagnosis, and treatment options of endometriosis, this paper covers the role of exosome lncRNAs in endometriosis and related molecular mechanisms.

## 1 Introduction

Exosomes are microsomal vesicles with a diameter of 30–160 nm produced by cells and control the transmission of information from cells to the extracellular matrix by carrying proteins, lncRNA, DNA, and other molecules (1–3). LncRNAs are RNAs longer than 200 nucleotides without the ability to code for proteins (4). lncRNAs have a significant role in several crucial regulatory processes, including nuclear transport, chromatin silencing, genomic imprinting, chromatin remodeling, transcriptional activation, and transcriptional interference (5, 6). LncRNAs play an essential role in the development of endometriosis, an estrogen-dependent condition in which endothelial cells in ectopic lesions are controlled by estrogen, as well as cancers, cardiovascular disorders, and hematologic diseases (7–9). It is a common gynecological, endocrine condition that harms women’s physical and emotional health (10–12). Exosome lncRNAs have been demonstrated to involved in the genesis of endometriosis, govern the activity of endothelium cells, and serve as a clinical marker for endometriosis (13, 14). They have also been linked to infertility in endometriosis patients. Compared to patients with stage I/II endometriosis and non-endometriosis, patients with stage III/IV endometriosis had significantly higher levels of TC101441 expression in their serum EVs. Because TC101441 can travel through EVs and control ESC migration and invasion, its presence in serum EVs may serve as a biomarker for endometriosis. Laparoscopy is the only current confirmation available and that many women may not be able to afford the procedure and this could be a cheap screen.

## 2 Overview of exosome

### 2.1 Biogenesis of exosome

**Figure 1:**
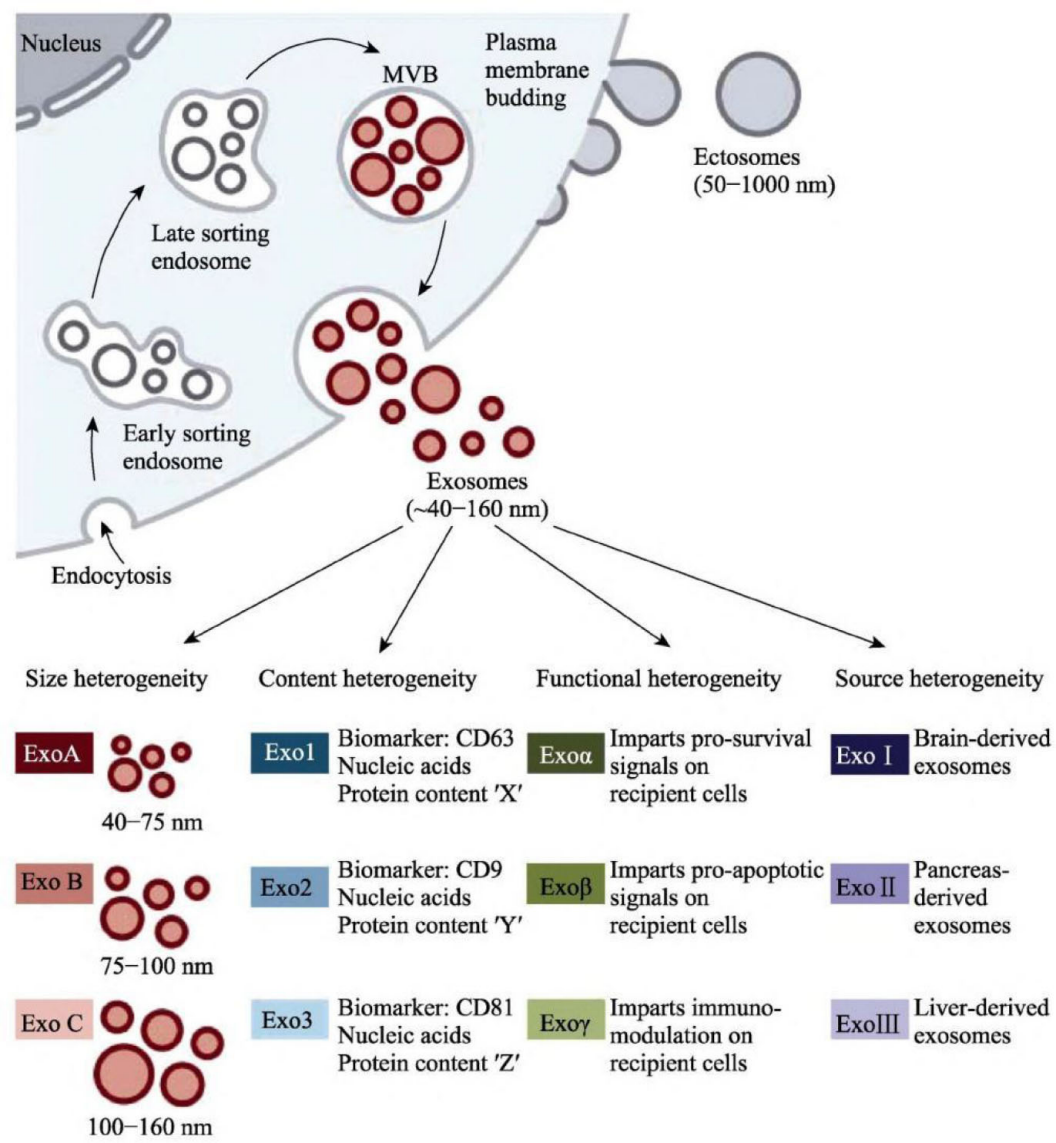
Production and secretion of exosomes (23).

Exosomes, which can be seen as cup-shaped objects under electron microscopy, are vesicles actively produced extracellularly by various live cells (15–17). The cell membrane invaginates to generate endosomes, multiple endosomes merge to form Early-Sorting Endosomes (ESE), and then ESE invaginates once more to wrap intracellular material and form numerous vesicles, which further convert into Late-Sorting Endosomes (LSE) (18, 19). Several tightly controlled mechanisms are involved in producing, sorting, and releasing the exosome’s contents. Several molecules, including the Endosomal Sorting Complex Needed for Transport (ESCRT), four transmembrane proteins (CD9, CD63, and CD81), the apoptosis-linked gene 2-interacting protein X (*Alix*), and the tumor susceptibility gene 101(*TSG101*), are involved in the intracellular transport of the exosome (20–22). The soluble N-ethylmaleimide-sensitive factor attachment protein receptor (SNARE) protein complex and the family of synaptic binding proteins are necessary for the production of exosomes (**Figure 1**) (23).

### 2.2 Exosome features

Exosomes are vital in transmitting materials and information between cells (24). They can reach the appropriate target cells or organs by direct fusion, endocytosis, receptor-ligand interaction, and other mechanisms (25, 26). Intercellular communication and biological processes like antigen presentation, immunological response, and cell differentiation are regulated by endometriosis in the body (27, 28). Endometriosis are involved in intercellular communication activities and govern biological processes, including antigen presentation, immunological response, and cell differentiation, thereby partaking in the initiation and progression of diseases (2, 29, 30). Proteomics analyses have shown exosomes from different cellular origins are heterogeneous (31, 32). Exosomes can physiologically affect many cells and tissues because of their various contents (33, 34). Exosomes from a healthy microenvironment may help preserve the target cells’ function. Still, exosomes from a stressful microenvironment might cause damage by sending oxidative and inflammatory signals to the target tissues (35). So, it is suggested that exosomes serve as significant intercellular communication carriers and can transport bioactive chemicals from their source cells, which may be crucial for intercellular communication (36–38).

### 2.3 Exosome extraction

While exosomes represent a potential tool for endometriosis diagnosis, their efficient purification without cellular contamination is a limiting factor (39). The primary techniques for isolating exosomes are gradient density centrifugation, differential ultracentrifugation, polymer immunoprecipitation, gel exclusion separation, and membrane affinity kits. One of the most popular separation techniques nowadays is ultracentrifugation, and the purest separation can be achieved using ultracentrifugation in combination with sucrose gradient density centrifugation (40). Exosomes have a low level of toxicity, are highly bioavailable, and are biologically stable (41). Consequently, it is anticipated that the identification and isolation of disease-specific exosomes free of cellular exosomes that are typically contaminated will shed new light on the advancement of precision medicine (42). Due to the size and physicochemical characteristics of exosomes, which differ from those of lipoproteins and protein complexes, as well as the fact that a variety of cells secretes exosomes, there is still much to learn about how to isolate and purify exosomes, which is a bottleneck problem in basic research and clinical applications related to exosomes.

## 3 Overview of lncRNA

### 3.1 Occurrence of lncRNA

LncRNAs are a family of RNAs with transcripts longer than 200 nucleotides frequently present in eukaryotic genomes but do not code for proteins (43, 44). Most lncRNAs can be found in the cytoplasm or nucleus of a cell and are produced from a single strand inside a protein-coding gene sequence (45). Antisense Bidirectional lncRNAs share promoters with protein-coding genes but are transcribed in the opposite direction to protein-coding genes; long intergenic ncRNAs (lincRNAs) are produced from the complementary DNA strand of the protein-coding gene, which is transcribed in the opposite direction and overlaps with at least one exon of the forward gene. Intronic lncRNAs are found in the protein-coding gene’s intron region and do not overlap with its exons (46, 47).

### 3.2 Biological functions of lncRNA

**Figure 2:**
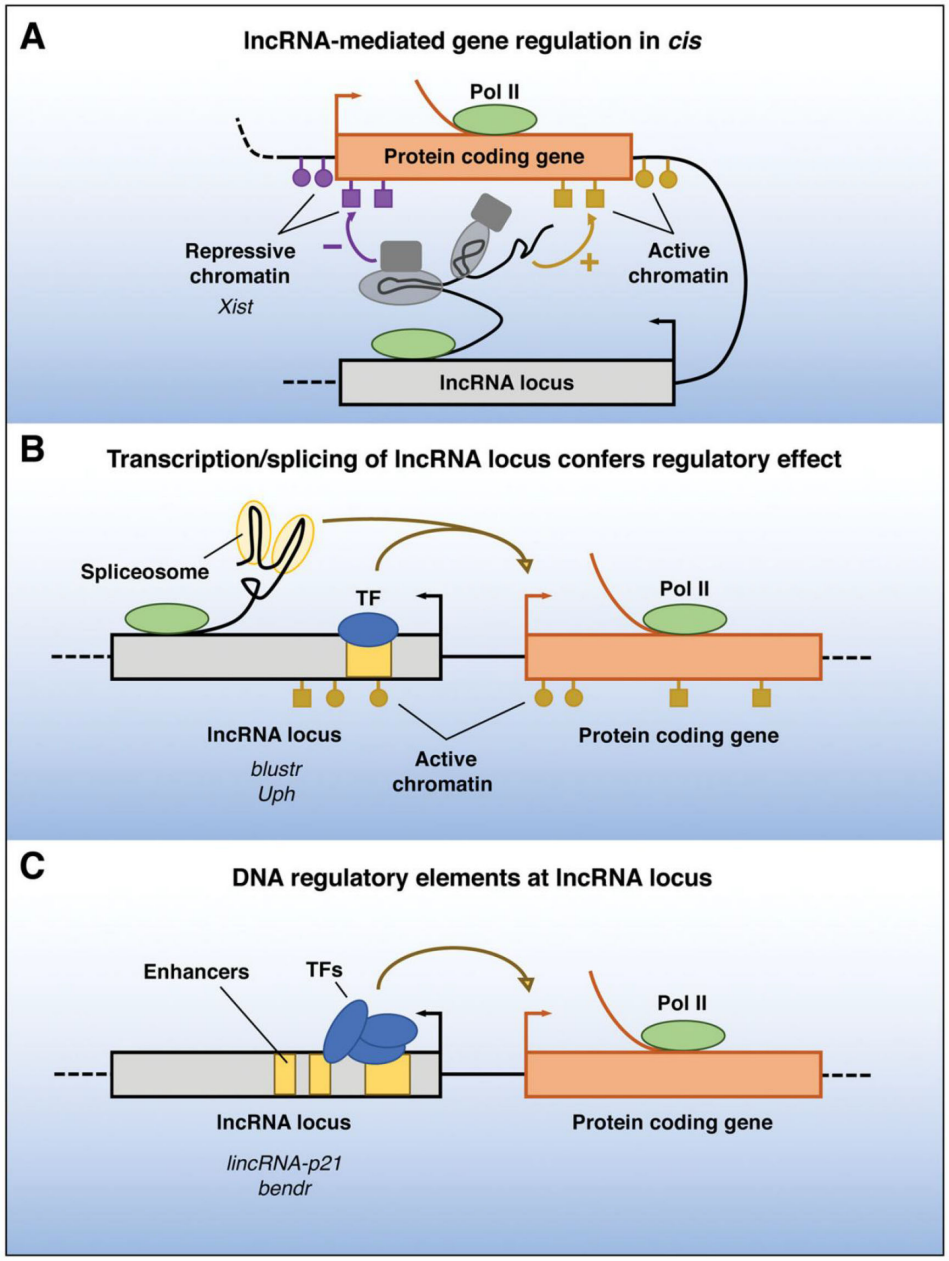
Functions of lncRNA loci in local gene regulation. The ability of a lncRNA locus to control the expression of nearby genes in cis may be due to DNA elements within the lncRNA promoter or gene body that function independently of the transcribed RNA **(A)**, may require transcription or splicing of an RNA but the lncRNA itself is not functional **(B)**, or may be due to sequence-specific functions of the mature lncRNA transcript **(C)**—Pol II, RNA polymerase II; TF, transcription factor (59).

Recent research has shown that lncRNAs are crucial for maintaining intracellular homeostasis when cells or tissues mature (48). This dispels the myth that noncoding RNAs (ncRNAs) are just “transcriptional noise” with no biological purpose (49, 50). It has been demonstrated that LncRNAs have a role in a variety of physiological and pathological processes (51, 52). *Via* epistasis, transcription, post-transcription, and acting as mediators of biological processes, lncRNAs are implicated in the regulation of apoptosis: epistasis lncRNAs can bind to Pol II to repress DNA expression, bind to DNA to form a triple helix structure to regulate, and can bind to transcription factors or recruit transcription-related factors to target genes to regulate the transcription of target genes (53–55). These lncRNA-specific structures or sequences can be specific sites on genomic DNA and can recruit the chromatin reconstruction complex (56, 57). The post-transcriptional regulation impacts the binding shear body and regulates the shearing process of mRNA by binding to antisense lncRNA at the target region of mRNA (**Figure 2**) (58, 59).

### 3.3 LncRNA regulates mRNA transport and translation

Before being translated into protein, pre-mRNA is specially spliced in the nucleus to generate mature mRNA, which is subsequently translocated from the heart to the ribosome in the cytoplasm. Only when the structural gene ultimately succeeds in producing an active protein is the gene considered functional (60). Several studies have demonstrated that lncRNAs can control mRNA translation in various ways (61). mRNA transport can be impacted by lncRNAs, which in turn control adipogenesis. In addition to acting as an insulin-sensitizing hormone to lower blood sugar and encourage adipogenesis, lncRNAs are an endogenous bioactive peptide released by adipocytes during lipogenesis. AdipoQ ASlncRNA joins forces with AdipoQ mRNA to create a double-strand in the nucleus, preventing AdipoQ mRNA from moving from the core to the cytoplasm suppressing adipogenesis. In order to play a part in controlling the stability of the target genes, lncRNA often attaches to the relevant mRNA or particular protein to form an RNA-RNA or RNA-protein complex (62). Antisense lncRNAs’ cytoplasmic binding to sense mRNAs is a typical example of this regulation. It has been demonstrated that lncRNAs can associate with RNA-binding proteins (RBPs) to create RNA-protein complexes that subtly control the stability of mRNA (63).

### 3.4 Competing endogenous RNA functions

LncRNAs can indirectly regulate the expression of target genes by competing for binding one or more miRNAs with the same response element, thereby promoting or inhibiting the onset or progression of disease (64, 65). This is a critical distinction between lncRNAs and miRNAs: the latter cannot interfere with or activate gene expression. LncRNAs can interact with miRNAs as a competitive endogenous RNA (ceRNA), functioning as a “sponge” for the miRNA molecule and easing its stress (66). This is one of the many ways lncRNAs and miRNAs function in cells. The ceRNA mechanism is a large and delicate method of gene expression control whose expression level is affected by conditions (67). LncRNAs, circRNAs, pseudogenes, synthetic miRNA inhibitors, and viral miRNA inhibitors are currently ceRNAs (68).

### 3.5 LncRNA controls the expression of inflammatory, immunological factors

LncRNAs can regulate gene expression and immune system responses, demonstrating biological processes’ tissue specificity and complexity (69). Involved in the body’s intrinsic and adaptive immunity, lncRNAs are crucial to many physical and immunological methods. The etiology of autoimmune and inflammatory illnesses is influenced by lncRNAs, such as lincRNA-Cox2 and lncRNA-dendritic cells (DC) (70). Numerous significant pathways connected to endometriosis are enriched in lncRNAs. Transient Receptor Potential (Trp) ion channels and thyroid hormone production were discovered to produce large amounts of inflammatory mediators, which suggests that lncRNAs may regulate the expression of inflammatory and immunological components linked to endometriosis (71). Inflammatory reactions may develop and be controlled in part by lncRNAs. According to immunological research, epigenetic dysregulation of B cells, which includes aberrant lncRNA expression, histone changes, and altered DNA methylation, might result in the body producing pathogenic autoantibodies. Endometriosis and autoimmune disorders share a biological mechanism with lncRNAs. Current hormonal and surgical treatments for endometriosis may be improved if the concept of immunomodulatory therapy for autoimmune illnesses is extended to the treatment of endometriosis (72, 73).

### 3.6 Correlation between lncRNA, miRNA, and endometriosis

Reverse regulatory RNA, siRNA competitive binding, and target gene stability regulation are techniques that LncRNAs can use to control target genes (74). LncRNAs can work as ceRNAs to control the expression of target genes, altering how well those genes function and becoming a key player in the emergence of disease (75, 76). MiR-199a can compete with vascular endothelial growth factor (VEGF)-A for the binding site on the lncRNA ENST00000465368, which reduces miR-199a’s ability to bind to its target gene VEGF-A and weakens miR-199a (77). Inducing downstream signaling pathways, endothelial cell proliferation and migration, increased microvascular permeability, promotion of neovascularization, and successful ectopic endothelial implantation are all made possible by the angiogenesis-specific regulator VEGF-A, which binds to its receptor (78, 79). A decrease in the expression of Syndecan-1 (*SDC1*), a cell adhesion molecule, could result from downregulating the expression of lncRNANR 033688. This could enhance the repressive effect of miR-10b on its target gene *SDC1*, which would reduce cell-cell and cell-matrix adhesion, facilitating the invasion of ectopic endometrial glandular epithelial cells (80). The growth of endometriosis and the interactions between miRNAs and lncRNAs are tightly connected, and endometriosis proliferation, invasion, and metastasis are all significantly impacted by these factors (81, 82).

## 4 Exosome lncRNA has excellent potential for clinical applications

Exosome-derived lncRNAs have potential uses. lncRNAs are RNAs with strong regulatory abilities (83). lncRNAs can control the expression of mRNA through ceRNA processes, bind directly to proteins to alter or increase their functions, form complexes with DNA to affect gene transcription, and more (84). One of the critical pathways in the development of endometriosis is lncRNA expression imbalance (85). The neural *CHL1* gene family includes the close homologue of the L1 (*CHL1*) gene, and CHL1-AS1 and CHL1-AS2 are two antisense lncRNA molecules of the CHL1 mRNA (86). Compared to *in situ* endometrial tissues, CHL1-AS1, CHL1-AS2, and *CHL1* mRNA expression was considerably higher in ectopic endometrial tissues. *CHL1* participates in the formation of endometriosis through interactions with CHL1-AS1 and CHL1-AS2. CHL1-AS1 or *CHL1* can bind to MiR-6076, which controls the expression of CHL1. Reduced expression of the CHL1-AS1 or increased miR-6076 have a protective effect on cell migration and proliferation (87).

Exosome lncRNA plays a significant role in the entire biological process of endometriosis compared to conventional endometriosis diagnosis and treatment. It exhibits the following features: Exosome lncRNA research helps ESCs proliferate, metastasize, and evolve while providing the molecular underpinnings for their targeted therapy. It is also more accessible for clinical diagnosis and relatively non-invasive (available in plasma, urine, and vaginal fluids) (88, 89). The exosome can influence the immune response in targeted therapy and release chemotherapeutic medicines and nucleic acids locally with low toxicity and high efficacy. Its contents are proteins and different nucleic acids uniquely expressed in distinct diseases. Exosome lncRNA research can investigate the pathophysiology of endometriosis, screen endometriosis for biomarkers, and offer a new fundamental framework for illness diagnosis and treatment (90).

## 5 Antifibrotic effects of exosome lncRNAs

The uterine cavity is invaded by endometrial cells, which then migrate to locations outside of it and undergo cyclic damage repair (91). The ability of stromal tissue to infiltrate and migrate causes a change from endometrial stromal fibrosis to fibrosis, which causes cell adhesion, collagen aggregation, and finally, fibrosis, resulting in the frequent presence of fibrous connective tissue around endometriosis lesions (92, 93). Endometrial stromal cells’ ability to transition from stromal fibrosis to fibrosis and the invasion and migration of endometrial cells can be aggravated by prolonged high estrogen stimulation levels in the body (94, 95). This can cause several clinical symptoms, including chronic inflammation, progressive pelvic pain, and infertility (96). Infertility is more difficult by the fibrosis forming the walls and surrounding ovarian endometriotic cysts (97). Under estrogenic control, highly expressed H19 can enhance actin alpha 2 (ACTA2) expression by decreasing miR-216a-5p, further boosting the invasive and migratory abilities of stromal cells and ultimately leading to the development of endometriosis lesions and the formation of fibrosis at the lesions (98, 99). A lncRNA called Homeobox Gene Transcript Antisense Intergenic RNA (HOTAIR) has been linked to several fibrotic disorders. By increasing miR-326, which decreases cell proliferation, promotes fibrosis, and promotes apoptosis, HOTAIR silencing may inhibit NUS1 expression. Exosome lncRNAs may develop into novel endometriosis inhibitory vectors and present novel therapeutic approaches for managing endometriosis. To treat fibrosis and aid in the prevention and treatment of endometriosis, antifibrotic lncRNAs carried by exosomes may be exploited as therapeutic targets.

## 6 Exosomal lncRNAs and endometriosis

### 6.1 Endometrial cells with tumorigenic properties

Endometrial glands and stromal implants, known as endometriosis, are found inside the uterine cavity but develop outside (100). Even though endometriosis is a benign condition, it is frequently described as “benign cancer” due to its local infiltration, implantation, metastasis, and recurrence (101–105). Surgery is an invasive procedure with some dangers, and pathological examinations take too long to diagnose endometriosis. More significant and of theoretical relevance are future investigations into the pathogenesis of endometriosis and the hunt for novel biomarkers and molecular targets for diagnosis and therapy (106). An essential characteristic of endometriosis is the enhanced capacity of endometrial cells to infiltrate and migrate (107). Endometriosis lesions adhere extensively or densely to the tissues around them, and many different lesions exist. lncRNAs play a significant role in controlling human disease, physiology, and cancer (108, 109). As elaborated in section 6.3, more researchers are discovering that lncRNAs are abnormally expressed in endometriosis patients and play a role in controlling the progression of the illness due to the in-depth study of lncRNAs in recent years. Exosome extracellular nucleotidase was discovered to be a diagnostic sign for endometriosis in ovarian endometriosis cysts.

### 6.2 Immunomodulatory imbalance is closely associated with the occurrence of endometriosis

**Figure 3:**
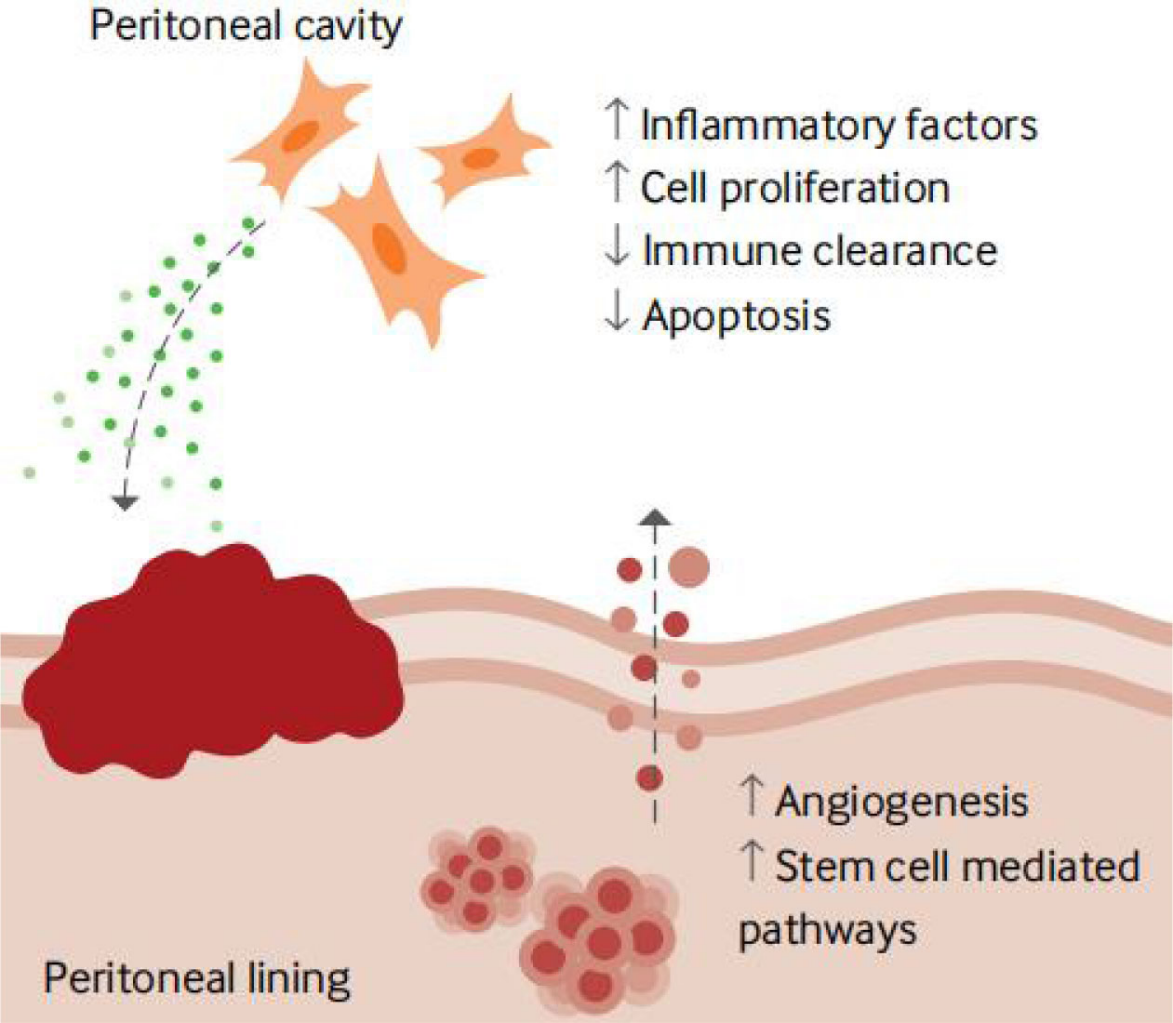
Local factors involved in developing an endometriosis lesion (111).

**Figure 4:**
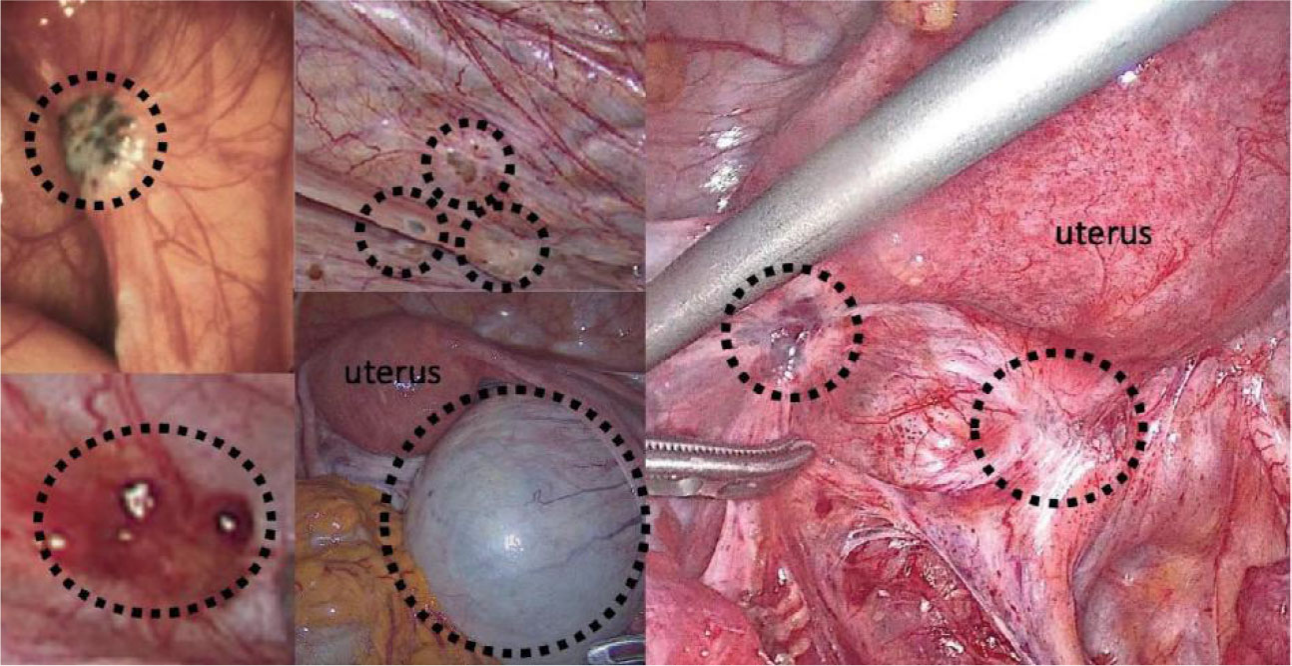
Surgical images of endometriosis sub-phenotypes (111).

The pathogenesis and pathophysiology of endometriosis, classified as chronic inflammatory illnesses, are significantly influenced by immunological variables. However, only a tiny portion of the population has endometrial fragments that spread, grow, and eventually develop into endometriosis. This is likely because of changes in the internal environment of endometriosis patients, such as immune imbalance, and the ectopic lesions are typically accompanied by inflammatory congestion and inflammatory infiltration (110). Many women experience the “menstrual reflux” phenomenon multiple times. One of the clinical classification indicators of endometriosis is the degree of inflammation. The abnormal immune function in endometriosis patients is primarily exhibited in two ways: on the one hand, it is a compromised immunosurveillance and immunocidal cytotoxic effect, which prevents the body from effectively clearing the endothelium; and on the other hand, it is a compromised immune environment in the abdominal cavity, which interferes with pregnancy and causes spots. Infertility, repeated implantation failure, early miscarriage, and aberrant histogenesis are related to endometriosis worsened by the peripheral circulating immune system and female endometrial autoimmune imbalance (**Figures 3**, **4**) (111).

### 6.3 Exosome lncRNA regulates the development of endometriosis

#### 6.3.1 Promotes neovascularization in endometriosis

Research has shown that neuro angiogenesis is crucial for the growth of endometriosis and that ESC-derived exosomes promote angiogenesis *in vitro* (112). Local angiogenesis and neurogenesis are encouraged, macrophages are activated, endometriosis lesions are made more extensive, and endometriosis advance when *in situ* ESC-derived exosomes are present (113). Angiogenesis-related lncRNA antisense hypoxia-inducible factor (a-HIF) is widely known (114). In the ectopic endometrium and serum of endometriosis patients, aHIF is abundantly expressed. Hence, exosome lncRNA-aHIFs are being investigated as potential molecular markers for the detection of endometriosis (115, 116). a-HIF plays a role in increasing angiogenesis in endometriosis and is strongly expressed in the endometriotic stromal cells (ECSCs) of patients with endometriosis. Another hypothesis for a molecular marker for the diagnosis of endometriosis is a-HIF. By activating VEGF-A, VEGF-D, and primary fibroblast growth factor (bFGF) to induce angiogenesis in human umbilical vein endothelial cells(HUVECs), exosomeaHIF is transported from ECSCs to HUVECs, which may have diagnostic relevance by stimulating angiogenesis in endometriosis (117, 118). The basic idea for the pathogenesis of endometriosis is the transcatheter blood flow reversal theory, and it is plausible that exosomes generated by healthy endometrium might travel through the blood to the pelvis or other places to advance endometriosis (119).

#### 6.3.2 Regulating the biological behavior of endometriosis cells

The emphasis of the research is on how exosome lncRNA affects the biological function of endometriosis. LINC00261 directly binds to miR-132-3p to regulate BCL2L11 expression, which inhibits the proliferation and invasion of endometriosis cells, suggesting that LINC00261 plays an inhibitory role in the occurrence and development of endometriosis (120, 121). Expression of LINC00261 was significantly downregulated in ectopic endothelial tissues. Cell proliferation and migration decreased considerably after LINC00261 was overexpressed. Since LINC01279 expression in endometriosis tissues was markedly increased and connected with cyclin-dependent kinase 14 (CDK14) expression, LINC01279 may control endometriosis’ cell cycle (122). In patients with endometriosis, lncRNACCDC144NL-AS1 showed differential expression in matched *in situ* and ectopic endothelial tissues. ESC migration and invasion were hindered by CCDC144NL-AS1 deletion, and CCDC144NL-AS1 knock-down reduced wave protein and matrix metalloprotein (MMP-9) expression, indicating that CCDC-144NL-AS1 may play a role in the pathogenesis of endometriosis (123). Application of BRAF-activated noncoding RNA (BANCR) inhibitors in a rat model of endometriosis resulted in a significant reduction in lesion size, accompanied by a decrease in mesenchymal cells and blood supply, and the BANCR inhibitors may inhibit endometriosis by inhibiting the extracellular signal-regulated kinase/mitogen-activated protein kinase (ERK/MAPK) signaling pathway to reduce the expression of molecules associated with angiogenesis (124).

#### 6.3.3 Correlation of cellular autophagy with the occurrence of endometriosis

Cellular autophagy is induced by hypoxia, a crucial microenvironmental element in endometriosis growth of endometriosis (125). Hypoxia caused a time-dependent induction of autophagy and the lncRNA metastasis-associated lung adenocarcinoma transcript 1 (MALAT1) in cultured human ESCs (126). nuclear factor kappa-B/induciblenitricoxidesynthase (NF-κB/iNOS) co-stimulation was linked to an inflammatory response, and increased expression of iNOS promotes angiogenesis (127). MALTA1 expression was considerably raised in ectopic tissues of patients with endometriosis. MALAT1 regulated the proliferation, invasion, and death of ESCs *via* NF-B/iNOS. On the one hand, the hypoxic environment in the pelvis boosted MALAT1 expression, while on the other, it promoted autophagy in ESCs, allowing endothelium debris to survive ectopically. And by inhibiting the autophagy brought on by hypoxia, MALAT1 expression suppression in ESCs may enhance apoptosis (126). Actinfilament-associatedprotein1-antisenseRNA1 (AFAP1-AS1) was significantly up-regulated in endometriosis tissues, and lncRNA-AFAP1-AS1 significantly downregulated the expression of pGL3-P886, the promoter of the transcription factor zinc-fingerE-boxbindinghomeobox1(ZTB1), promoted epithelial-mesenchymal transition (EMT), and increased ectopic endometrial invasion and implantation (128). By encouraging EMT, LINC01541 can be silenced to improve ESC invasion, whereas LINC01541 overexpression can inhibit EMT, ESC metastasis, and VEGF-A production, as well as cause apoptosis through controlling the Wnt/-catenin pathway (129). Exosome lncRNA encourages endometriosisESC invasion and proliferation, and it can act as a diagnostic marker for endometriosis to aid in the early detection of endometriosis.

### 6.4 Exosome lncRNA mediates infertility linked to endometriosis

Patients with endometriosis frequently experience infertility, and aberrant changes in endometrial status and uterine artery flow resistance can impact endometrial tolerance and, in turn, affect pregnancy and prognosis (130–132). Studies have revealed that the *in situ* endometrium of endometriosis exhibits a marked reduction in H19 expression (133). H19 usually is abundantly expressed in late-proliferating ESCs, whereas in the situ endometrium of endometriosis, its expression is downregulated. Low levels of H19 raise the activity of miRNALet-7, which in turn suppresses *in situ* ESC proliferation and survival and inhibits the downstream target insulin-like growth factor 1 receptor (IGF1R) at the post-transcriptional level. This results *in situ* endometrial defects, which affect endometrial tolerance and obstruct embryo implantation. By interfering with the H19/Let-7/IGF1R regulation system, endometriosis patients may experience spontaneous miscarriage or infertility due to diminished endometrial readiness and pregnancy receptivity. Through controlling miR-124-3p and Integrin beta-3 (ITGB3), lncRNA-H19 downregulation may prevent the proliferation and invasion of ectopic endometrial cells. This gives the treatment of endometriosis a new target (134).

Endometriosis can hinder oocyte growth and maturation, and as endometriosis levels rise, so does the disruption of ovarian reserve function. The expression of lncRNMALAT1 is increased in ectopic endometrial tissue (135). Through increasing the EMT-related transcription factors ZEB1 and ZEB2, MALAT1, a ceRNA of miR-200C, can support ESCs, boosting proliferation, invasion, and ectopic pregnancy. In ovarian granulosa cells of endometriosis, the expression of lncRNA-MALAT1 was markedly downregulated and positively associated with the number of sinus follicles (136). Further *in vitro* research supported the hypothesis that MALAT1 knock-down could affect follicular granulosa cell proliferation by activating the ERK/MAPK signaling pathway and up-regulating the expression of cell cycle regulatory proteins and that up-regulating the lncRNAENST00000433673 could affect embryo implantation and impair female fertility (137–139).

### 6.5 A potential diagnostic sign for endometriosis is exosome lncRNA

Exosome lncRNAs have proven to offer promise as non-invasive biomarkers for determining the presence of illness. In endometriosis, lncRNA AC002454.1 is considerably overexpressed, and the expression level is closely associated with cyclin-dependent kinase6(CDK6) levels (140). The biological behavior of endometrial cells may be cooperatively influenced by AC002454.1 and CDK6, which may work in concert to encourage the growth of endometriosis (141). In the endometrial tissues of endometriosis patients and healthy individuals, 86 lncRNAs showed differential expression. KEGG pathway analysis revealed that these lncRNAs were connected to various biological functions and signaling pathways in endometriosis (142). The lncRNA ENST00000482343 in the serum of endometriosis patients could be used as a potential molecular marker, according to earlier studies that examined the differential expression profiles of lncRNAs in 110 serum samples and 24 tissue samples and assessed their diagnostic value using the receiver operating characteristic curve. In ovarian endometriotic tissues, Urothelial Cancer Associated 1 (UCA1) expression was markedly downregulated, and the extent of UCA1 downregulation was positively connected with the severity of ovarian endometriosis (143). UCA1 might be used as a biomarker to evaluate the prognosis and staging of ovarian endometriosis (143). HOXA11-AS1 and *HOXA* gene mRNA fragments Patients with endometriosis had considerably lower expression levels in their *in situ* endometrium than in their ectopic endometrium, which raises the possibility that HOXA11-AS1 may be involved in the growth of peritoneal endometriosis (144).

In comparison to *in situ* and normal endometrium, ectopic endometrium expressed TC0101441 at a greater level. High expression of TC0101441 in ECSCs produced EVs, which were subsequently internalized and absorbed by low-expression ECSCs to achieve TC0101441 transfer in ECSCs and eventually encourage endometriosis migration and invasion (145). Women with endometriosis had less maternally expressed gene 3 (MEG3)-210 in their ectopic endometrium (146). ESC migration and invasion are encouraged by the downregulation of MEG3-210. Through interacting with Galectin-1 *via* p38MAPK and cyclic AMP-dependent protein kinase A/sarco-endoplasmic reticulum ATPase (PKA/SERCA) signaling, mEG3-210 promotes ESC migration and invasion (147). In endometriosis, miR-145 reduces tumor growth, invasiveness, and stem cell characteristics. Prostate cancer-associated transcript-1 (PCAT-1) lncRNA and siRNA dramatically boosted miR-145 expression, reducing endometriotic stem cell proliferation and invasiveness (148). Normal endometrial tissues had a lower ratio of lncRNA steroid receptor activator (SRA) to steroid receptor activating protein (SRAP) than endometriosis tissues. In contrast to normal endometrial tissue, the expression of SRA and ER-lncRNA in endometriotic ovarian tissue was lower than that of SRAP and ER-lncRNA. Through controlling ER, SRA in ovarian endometriosis may play a significant regulatory function in the development of ESCs (149).

## 7 Conclusion

Exosome lncRNA can be a valuable biomarker for endometriosis, a prevalent gynecological, endocrine disorder that harms women’s reproductive health and significantly burdens society. The present diagnostic indicators for endometriosis have low specificity and sensitivity. Hence, alternative diagnostic indicators must be discovered to increase the diagnostic effectiveness of endometriosis. lncRNAs are crucial for epigenetic control, transcription, translation, RNA metabolism regulation, cell autophagy, and death, among other processes. LncRNAs are quickly disturbed by other components in the circulation; however, because of the protection provided by the EVs structure, lncRNAs encapsulated in exosomes are not as easily destroyed once they have entered the circulation. Exosome lncRNA is stably expressed in serum or bodily fluids and can be employed as a disease biomarker to create a diagnostic model that will direct early diagnosis and postoperative follow-up of endometriosis, enable early disease treatment, and avoid recurrence. Exosome lncRNA research in endometriosis is still in its infancy, and there are still a lot of unexplored frontiers. The issues mentioned above can be resolved piecemeal with the rapid advancement of proteomics, transcriptomics, and bioinformatics analysis. Researchers will have a better knowledge of the role of exosome lncRNA in the genesis of endometriosis and its therapeutic utility.

## Author contributions

MW, LZ, RL, SM, JL, and SY performed literature searches and selected the studies and reviews discussed in the manuscript. The first draft of the manuscript was prepared by MW. LZ, RL, SM, and JL made subsequent amendments. SY revised the manuscript. All authors contributed to the article and approved the submitted version.

## Glossary

**Table:**

EVs	extracellular vesicles
lncRNA	long noncoding RNA
ESE	early-Sorting Endosomes
LSE	Late-Sorting Endosomes
ESCRT	Endosomal Sorting Complex Needed for Transport
Alix	apoptosis-linked gene 2-interacting protein X
TSG101	tumor susceptibility gene 101
SNARE	soluble N-ethylmaleimide-sensitive factor attachment protein receptor
lincRNAs	long intergenic ncRNAs
ncRNAs	noncoding RNAs
Pol II	RNA polymerase II
TF	transcription factor
RBPs	RNA-binding proteins
ceRNA	competitive endogenous RNA
DC	dendritic cells
Trp	Transient Receptor Potential
VEGF	vascular endothelial growth factor
SDC1	Syndecan-1
CHL1	close homologue of the L1
ACTA2	actin alpha 2
HOTAIR	Homeobox Gene Transcript Antisense Intergenic RNA
aHIF	antisense hypoxia-inducible factor
ECSCs	endometriotic stromal cells
bFGF	basic fibroblast growth factor
HUVECs	human umbilical vein endothelial cells
CDK14	cyclin-dependent kinase14
MMP-9	matrix metalloprotein-9
BANCR	BRAF-activated noncoding RNA
ERK/MAPK	extracellular signal-regulated kinase/mitogen-activated protein kinase
MALAT1	metastasis-associated lung adenocarcinomatranscript 1
NF-κB/iNO	nuclear factor kappa-B/inducible nitricoxide synthase
AFAP1-AS1	Actinfilament-associatedprotein1-antisenseRNA1
ZTB1	zinc-fingerE-boxbindinghomeobox1
EMT	epithelial-mesenchymal transition
IGF1R	insulin-like growth factor 1 receptor
ITGB3	Integrin beta-3
CDK6	cyclin-dependent kinase6
UCA1	Urothelial Cancer Associated 1
MEG3	maternally expressed gene 3
PKA/SERCA	cyclic AMP-dependent protein kinase A/sarco-endoplasmic reticulum ATPase
PCAT-1	Prostate cancer associated transcript-1
SRA	steroid receptor activator
SRAP	steroid receptor activating protein
